# Suspected Xylazine-Associated Skin Ulcers in Montreal, Canada: A Case Report on Diagnostic Challenges and Multidisciplinary Care

**DOI:** 10.7759/cureus.114613

**Published:** 2026-08-16

**Authors:** Paris Dastjerdi, Samah Ibrahim

**Affiliations:** 1 Psychiatry, McGill University, Montreal, CAN

**Keywords:** canada, drug adulteration, fentanyl, harm reduction, necrosis, skin ulcer, substance use disorder (sud), tranq, tranq wounds, xylazine

## Abstract

Xylazine is a non-opioid α2-adrenergic receptor agonist originally synthesized as an antihypertensive but repurposed for veterinary sedation due to severe central nervous system side effects. In recent years, xylazine has increasingly entered the North American illicit drug supply, frequently adulterating stimulants and opioids to prolong and intensify their psychoactive effects. Its potent α2-adrenergic agonist activity has been proposed to contribute to localized skin ischemia and necrotic wound development via peripheral vasoconstriction, though the exact pathogenesis remains incompletely understood and is likely multifactorial. This report documents what is, to our knowledge, the first reported Canadian case of suspected xylazine-associated skin ulceration in the peer-reviewed literature. We present the case of a 64-year-old male with a history of polysubstance use who was admitted to a tertiary care hospital in Montreal, Quebec, on two separate occasions with severe, treatment-resistant necrotic ulcerations across his extremities. Standard therapeutic interventions, including multiple antibiotic courses and surgical debridement, yielded no clinical improvement. Consultation-liaison psychiatry evaluation uncovered that the patient was actively using illicit cocaine during his hospitalization, raising clinical suspicion for xylazine contamination of his drug supply. Confirmatory xylazine testing was not available within the relevant detection window; accordingly, the diagnosis is clinical and presumptive, based on characteristic wound features, temporal association with use of an illicit drug supply known to contain xylazine, and exclusion of alternative etiologies. Following targeted psychoeducation, substance cessation, and a multidisciplinary wound management strategy, the patient's wound progression halted and significant healing was observed. This case highlights critical diagnostic and public health challenges, including limited clinician familiarity with xylazine-associated wounds, insufficient rapid screening infrastructure, and the absence of standardized clinical management guidelines in Canada. Frontline healthcare providers should maintain a high index of suspicion for xylazine exposure when evaluating non-healing, progressive necrotic lesions in individuals who use illicit substances.

## Introduction

Xylazine is a non-opioid α2-adrenergic receptor agonist synthesized in 1962 as an antihypertensive; however, due to severe CNS side effects, it was repurposed for veterinary sedation [1]. In North America, xylazine entered the illicit drug supply in the early 2000s [2] and has increasingly been added to stimulants and opioids to prolong and intensify psychoactive effects [3].

In Canada, xylazine emerged as a public health concern first in 2019 [4]. A 2025 Health Canada update, based on data from 11 participating Canadian provinces and territories, reported that xylazine identifications in seized samples were nearly 14-fold higher in 2024 than in 2019. Concurrently, xylazine-related toxicity deaths increased approximately 50-fold, rising from 0.1 per 1,000,000 population (five deaths) in 2019-2020 to 4.8 per 1,000,000 population (90 deaths) in 2024. These statistics reflect deaths in which xylazine was specifically documented on the medical death certificate as a contributing cause of death and do not include granular toxicological data [5]. Although the overall population-level incidence remains relatively low, these findings represent a sharp upward trajectory in xylazine-related mortality.

Xylazine's potent α2-adrenergic agonist activity has been proposed to contribute to localized skin ischemia and necrotic wound development via peripheral vasoconstriction. However, the exact pathogenesis of xylazine-associated cutaneous lesions is not completely understood and is likely multifactorial. Contributing factors may include repeated injection-related trauma, chemical contaminants within the illicit drug supply, bacterial colonization, compromised nutritional or immune status, underlying vascular disease, and delayed medical presentation [6,7]. These ulcers can begin as small distal skin lesions and rapidly progress to deep wounds with exposed fascia, tendon, or bone, sometimes leading to osteomyelitis or amputation [8]. Although these ulcerations are predominantly documented among individuals who inject substances, frequently manifesting at non-injection sites distant from direct venipuncture marks, emerging reports have also described similar lesions via non-injection routes (e.g., inhalation or insufflation), though the evidence base for non-injection modalities remains limited [6]. Because xylazine-associated ulcers lack pathognomonic histological or clinical features, they remain a diagnosis of exlusion, requiring careful clinical differentiation from mimicking conditions, such as necrotizing soft tissue infections, pyoderma gangrenosum, vasculitis, calciphylaxis, arterial insufficiency, and atypical infections [6].

Though cases have been reported in the US since 2022 [9-16], this is, to our knowledge, the first documented Canadian case highly suspicious for xylazine-associated ulceration in the literature. Confirmatory xylazine testing was not available within the relevant detection window; accordingly, the diagnosis in this case is clinical and presumptive, based on characteristic wound features, temporal association with use of an illicit drug supply known to contain xylazine, and exclusion of alternative etiologies. This report highlights challenges in diagnosis, management, and opportunities for harm reduction policies.

Written informed consent was obtained from the patient for the publication of this case report, including clinical details and images.

## Case presentation

A 64-year-old man with a history of intravenous (IV) fentanyl and heroin use presented to the emergency department of a tertiary university hospital in Montreal, Canada. Several days prior, erythematous pustules had developed on his right fingers, spreading proximally to his mid-humerus. On physical examination, there were no acute venipuncture marks, track marks, or localized injection stigmata on either upper extremity. The patient reported past IV substance use but endorsed sustained IV abstinence, reporting that his recent drug exposure was restricted to occasional smoked cocaine prior to admission. Notably, at presentation, the patient did not receive immediate toxicology screening or an early psychiatric consultation to objectively verify or evaluate his substance use history.

On exam, the right forearm had a painful, serous-draining ulcer with honey-colored crusting and smaller open wounds on the fingers. The arm was swollen, erythematous, and showed non-pitting edema and induration extending over 4 cm beyond the wound. The skin was dark red to purple and non-blanchable. Range of motion was reduced. The wound bed was 50-75% epithelialized with diffuse, indistinct edges. He was afebrile and hemodynamically stable.

CT showed subcutaneous edema and skin thickening consistent with cellulitis. He was admitted for presumed cellulitis and bacteremia, started on empiric IV antibiotics, and received wound care every three days (saline irrigation, Iodoflex with silver, Mepilex foam, and Kling wrap). He improved and was discharged after 12 days with oral antibiotics.

Several months later, he returned with similar but more severe symptoms in his left leg. Swelling and redness had begun three weeks earlier and spread to the thigh. He reported purulent discharge from the shin.

On exam, the left leg had irregular, coalescing ulcers with full-thickness skin loss, exposed muscle, and slough (Figure 1). The surrounding skin was dark red to purple. Redness extended to the scrotal region, but genitalia were unaffected. He denied trauma, insect bites, or wounds. As with his initial presentation, no formal toxicology screening was performed, and psychiatry was not consulted at presentation. 

**Figure 1:**
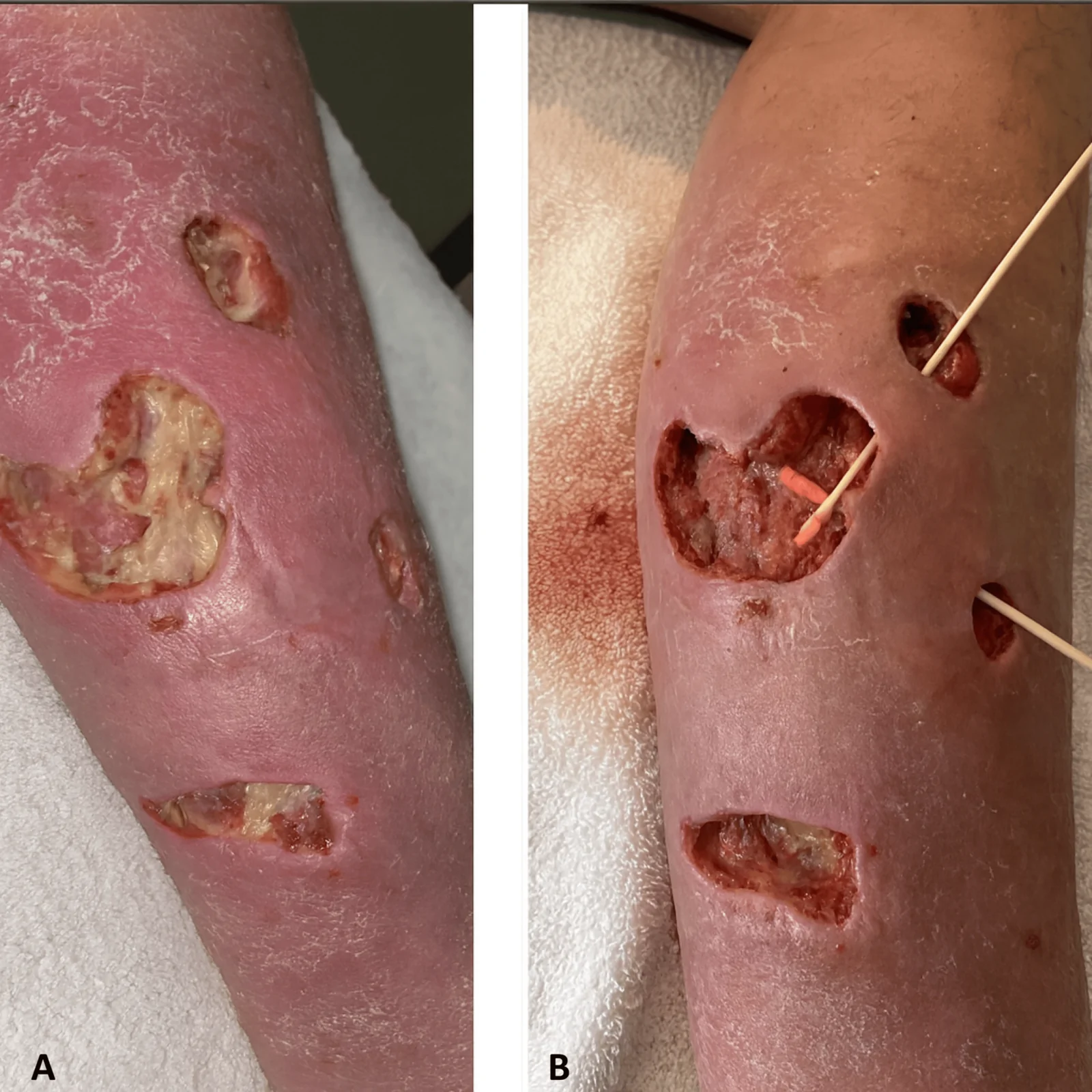
Clinical progression of suspected xylazine-associated cutaneous ulceration on the patient's left lower extremity. (A) Left lower extremity presentation upon initial hospital admission, demonstrating progressive tissue ischemia and extensive necrotic ulceration prior to surgical debridement. (B) Left lower extremity following surgical debridement, demonstrating clean wound edges, reduction of devitalized tissue, and initiation of a structured wound-healing framework.

He was admitted to the hospital and underwent two bedside debridements, daily wound care, and multiple IV antibiotic courses. No improvement was noted. Various consultations and investigations were performed while hospitalized. Consultation services included immunology and infectious diseases, plastic surgery, orthopedic surgery, dermatology, and vascular surgery. Investigations performed included autoimmune and infectious panels, Doppler, ankle-brachial index (ABI) testing, and skin biopsy, none of which clarified the diagnosis.

By hospital day 29, the patient was warned regarding a potential amputation if the wounds continued to progress. This became a source of profound emotional distress for the patient, and the consult-liaison psychiatry service was subsequently consulted. During psychiatric evaluation, the patient disclosed that visitors had been bringing him cocaine during hospitalization to cope with his stress. Given the clinical course, rapid wound progression, and lack of response to antibiotics, and in the light of the information the patient shared regarding ongoing cocaine use, xylazine-associated ulceration was suspected by the psychiatry team.

With his consent, psychiatry requested xylazine testing. Initial hospital lab inquiries yielded confusion as this was a test the hospital lab was not aware of. Eventually, the Centre de Toxicologie du Québec (CTQ) confirmed that testing was possible, requiring a special collection tube and send-out protocol. However, the patient had already abstained from drug use following extensive psychoeducation and the suspected relationship between xylazine and his ulcers, and with xylazine's short detection window, CTQ advised that testing would not be helpful.

Within days of substance cessation and continued wound treatment and antibiotics, the aggressive peripheral progression of the necrosis stopped, giving way to healthy granulation tissue and significant marginal contraction. By hospital day 39, exactly 10 days after the initial psychiatric consultation, the acute risk of amputation was successfully averted, and the limb was stabilized.

## Discussion

This case report documents what is, to our knowledge, the first reported Canadian case of suspected xylazine-associated ulceration in the peer-reviewed literature. To support this claim, a comprehensive literature search was conducted across PubMed, Embase, and Google Scholar from database inception through August 2026. The search combined the term "xylazine" with skin-related terms ("skin ulcer," "wound," "necrosis," "skin lesion") and Canadian geographic identifiers, including "Canada," "Canadian," and all provincial names (Ontario, British Columbia, Alberta, Quebec, Manitoba, Saskatchewan, Nova Scotia, New Brunswick, Newfoundland, Prince Edward Island), as well as major Canadian cities (Toronto, Vancouver, Calgary, Halifax and Montreal). Reference lists of all identified articles were hand-searched for additional relevant publications. No prior Canadian cases of suspected xylazine-associated skin ulceration were identified. This finding was further corroborated by a recent scoping review by Vaile et al., which systematically searched PubMed and Scopus, screening 2,711 articles and identifying 10 studies meeting inclusion criteria for xylazine-associated wounds, none of which originated from Canada [7]. These findings support the novelty of the present report.

Xylazine-associated ulcers are typically rapidly progressive, necrotic, and profoundly resistant to conventional antibiotic courses. Consistent with existing literature from Philadelphia [9-12], Florida [13-15], and Connecticut [16], this patient's ulcers developed across multiple extremity sites without evidence of local injection trauma. This distinct physiological presentation reinforces the clinical necessity to suspect xylazine exposure in any patient using illicit substances, such as cocaine, heroin, or fentanyl, who presents with atypical, non-healing necrotic wounds.

This case emphasizes the value of structured, interdisciplinary collaboration, including emergency medicine, psychiatry, infectious diseases, orthopedic surgery, dermatology, and specialized wound care, in managing complex xylazine-associated ulcers. An important turning point in this patient's care came with psychiatric involvement, which uncovered the ongoing substance use and ultimately pointed the team toward the suspected diagnosis. A lack of clinical familiarity with these unique, adulterant-associated wounds contributes to prolonged hospitalizations and extensive, invasive investigations that could be entirely avoided with earlier recognition. Furthermore, because patients are frequently unaware of xylazine contamination in their supply, an accurate clinical diagnosis is vital to facilitate transparent patient communication and targeted psychoeducation.

Diagnosis is further hindered by the limited accessibility of toxicological testing. Xylazine screening is not included in standard hospital urine drug panels in Canada. Formal confirmation requires coordination with specialized provincial laboratories and must occur within a narrow detection window following consumption. A lack of staff familiarity with these specific ordering workflows often leads to missed windows for definitive detection.

Rapid response xylazine test strips, developed by a Canadian biotechnology firm (BTNX Inc.; the authors declare no conflicts of interest or affiliations with this company) and authorized by Health Canada, are designated for detecting xylazine in drug samples as well as human urine [17,18], with literature documenting their utility in harm reduction settings [14]. If integrated into Canadian healthcare settings, these strips could serve as a valuable, low-cost, point-of-care diagnostic asset to aid earlier recognition. Additionally, distributing these strips through community harm reduction networks, parallel to fentanyl test strip initiatives, could empower individuals who use substances to audit their supply and mitigate accidental exposure.

Currently, there are no national clinical guidelines in Canada for the medical management of xylazine-related wounds. Harm reduction organizations in the United States, such as Prevention Point Philadelphia, recommend treatment protocols based on the DIME framework (debridement, infection control, moisture balance, and periwound edge assessment), utilizing advanced dressings and enzymatic agents such as medical-grade honey or collagenase ointment alongside non-adherent layers [19]. Consistent, evidence-informed protocols are urgently needed across Canadian healthcare settings to optimize clinical outcomes, reduce morbidity, enable outpatient management through harm reduction partnerships, and facilitate patient education on self-directed wound care.

The clinical course of this patient illustrates the potential value of considering xylazine contamination in the differential when evaluating refractory necrotic wounds in patients suspected of substance use. Prior to suspecting the exposure, the patient's progressive necrosis had not responded to conventional medical therapies, and surgical amputation was being considered. Recognition of possible xylazine exposure prompted a shift in the treatment that incorporated psychiatric follow-up and substance cessation counseling. Wound improvement in this case occurred in the context of multiple concurrent interventions, including wound care, debridement, antibiotics, and inpatient multidisciplinary management; as such, the relative contribution of xylazine cessation cannot be isolated from these therapies in a single case report. However, emerging evidence suggests that discontinuation of xylazine-contaminated drug use may be independently associated with wound healing. Makhoul et al. reported that, among 66 surgical consultations for xylazine-associated wounds, patients who stopped substance use had higher full healing rates than those who continued (75.0% vs. 20.4%, p = 0.005), even when concurrent wound care and surgical interventions were provided [20]. Similarly, Vaile et al. noted that wound appearance improved when patients discontinued xylazine-adulterated opioids [7]. Although causation cannot be established from observational data alone, this case adds to the growing body of literature suggesting that clinical suspicion for xylazine-associated ulceration, even without confirmatory toxicological testing, may meaningfully inform management decisions and potentially contribute to improved outcomes.

## Conclusions

This case underscores the clinical importance of maintaining a high index of suspicion for presumptive xylazine-associated cutaneous lesions within the Canadian healthcare landscape. As xylazine increasingly adulterates the illicit drug supply, frontline healthcare providers should recognize these presentation patterns and evaluate them alongside critical differential diagnoses, including necrotizing soft tissue infections, pyoderma gangrenosum, vasculitis, calciphylaxis, arterial insufficiency, and atypical infections. While a single case without toxicological confirmation cannot establish definitive causality, our findings highlight the value of structured, multidisciplinary collaboration, combining specialized wound management with psychiatric and addiction medicine involvement, to support wound recovery and reduce amputation risk. Expanding access to point-of-care drug checking technologies, standardized wound care protocols, and harm reduction initiatives across Canadian healthcare settings may improve clinical recognition and patient outcomes.
